# GlSwi6 Positively Regulates Cellulase and Xylanase Activities through Intracellular Ca^2+^ Signaling in *Ganoderma lucidum*

**DOI:** 10.3390/jof8020187

**Published:** 2022-02-14

**Authors:** Ling-Dan Lian, Ling-Yan Shi, Jing Zhu, Rui Liu, Liang Shi, Ang Ren, Han-Shou Yu, Ming-Wen Zhao

**Affiliations:** Key Laboratory of Agricultural Environmental Microbiology, Ministry of Agriculture, Microbiology Department, College of Life Sciences, Nanjing Agricultural University, Nanjing 210095, China; 2017216026@njau.edu.cn (L.-D.L.); 2020216026@njau.edu.cn (L.-Y.S.); jingzhu@njau.edu.cn (J.Z.); ruiliu@njau.edu.cn (R.L.); shiliang@njau.edu.cn (L.S.); angren@njau.edu.cn (A.R.); yuhans@njau.edu.cn (H.-S.Y.)

**Keywords:** GlSwi6, cellulase, xylanase, Ca^2+^, *Ganoderma lucidum*

## Abstract

*Ganoderma lucidum* is a white-rot fungus that produces a range of lignocellulolytic enzymes to decompose lignin and cellulose. The mitogen-activated protein kinase (MAPK) pathway has been implicated in xylanases and cellulases production. As the downstream transcription factor of Slt2-MAPK, the function of Swi6 in *G. lucidum* has not been fully studied. In this study, the transcription factor GlSwi6 in *G. lucidum* was characterized and shown to significantly positively regulate cellulases and xylanases production. Knockdown of the GlSwi6 gene decreased the activities of cellulases and xylanases by approximately 31%~38% and 54%~60% compared with those of the wild-type (WT) strain, respectively. Besides, GlSwi6 can be alternatively spliced into two isoforms, GlSwi6A and GlSwi6B, and overexpression of GlSwi6B increased the activities of cellulase and xylanase by approximately 50% and 60%, respectively. Further study indicates that the existence of GlSwi6B significantly increased the concentration of cytosolic Ca^2+^. Our study indicated that GlSwi6 promotes the activities of cellulase and xylanase by regulating the Ca^2+^ signaling. These results connected the GlSwi6 and Ca^2+^ signaling in the regulation of cellulose degradation, and provide an insight for further improvement of cellulase or xylanase activities in *G. lucidum* as well as other fungi.

## 1. Introduction

*Ganoderma lucidum* is an important basidiomycete, and its secondary metabolites have attracted wide attention due to their anticancer and other pharmacological activities [1,2]. *G. lucidum* satisfies its growth and development requirements by degrading a variety of agro-industrial lignocellulosic biomass materials [3]. This degradation is achieved through the production of a series of lignocellulolytic enzymes that decompose lignin and cellulose [4]. Analysis of the *G. lucidum* genome sequencing results revealed that its genome encodes the most abundant group of wood degradation enzymes among all sequenced basidiomycetes [2]. These enzymes mainly digest plant cell wall polysaccharides such as cellulose, hemicellulose and pectin. In addition, analysis of presumed lignocellulose-related secretory proteins in *G. lucidum* also indicated that cellulose, hemicellulose, and lignin-degrading enzymes are necessary for the degradation of lignocellulosic substrates for the generation of biofuel [5]. In addition, the potential use of mushrooms as biotechnological sources of enzyme sources has attracted wide interest. Therefore, it is important to investigate the underlying regulatory mechanism of cellulose-related enzymes in mushrooms.

The lignocellulolytic enzymes produced by microbes are complex carbohydrate active enzymes (CAZy) that contain numerous types of degrading enzymes, which are categorized as cellulases, hemicellulases and lignin-modifying enzymes [6,7]. Among them, both cellulase and hemicellulose belong to the glycoside hydrolase (GH) family, while lignin-modifying enzymes belong to the auxiliary activity (AA) protein family. The GH family about cellulase includes exoglucanases/cellobiohydrolases (CBH), endoglucanases (EG) and beta-glucosidases (BGL) [8]. Hemicellulases mainly consist of xylanases (XLN) that decompose the xylan skeleton and its side chain [9]. In addition, transcriptional regulation of cellulolytic gene expressions have also been reported to play a crucial role in the control of carbohydrate hydrolysis processes. Several transcriptional factors of cellulose-degrading pathways have been identified in cellulolytic fungi. CreA/Cre1/Cre-1, AceI, AceII, Ace3, and AmyR were reported to play a negative role in the cellulase and xylanase regulatory network [10]. CreA/Cre1/Cre-1 is a regulator of carbon catabolite repression that plays a pivotal role in the activation or inhibition of cellulose deconstruction [11]. AceI, AceII, and Ace3 are activators of cellulase expression [12]. AmyR is a transcriptional activator involved in amylolytic gene expression, which have also been found to affect hemicellulolytic and cellulolytic enzyme production [13], while Xyr1/XlnR1/XlnR-1, ClrB/Clr-2/ManR, and BglR are reported to function as positive transcription factors [10]. Recently, several signaling pathways such as Ca^2+^ and MAPK have also been reported to have regulatory effects on the expression of cellulose-related enzymes. The addition of Ca^2+^ enhanced the cellulase production [14]. Wang’s study provided evidence that Ca^2+^ signaling participates in the regulation of cellulase production by increasing intracellular NAD^+^ content [15]. Furthermore, the MAPK pathway in ascomycetes are also reported to be involved in the regulation of cellulolytic enzyme synthesis. The Chk1 MAPK signaling pathway is induced by environmental signals to positively regulate the expression of two cellulase-encoding genes, CBH7 and EG6 in *Cochliobolus heterostrophus* [16]. However, few studies have investigated the involvement of signaling in cellulose degradation, and the associations of different signaling pathways involved in regulating cellulose and xylanase activities are also unclear.

Swi6, a member of the fungi-specific APSES transcription factor family, has been reported to be a key regulator of fungal biological processes [17,18]. Besides, Swi6 is also a target of Mpk1/Slt2 MAPK in *Saccharomyces cerevisiae* [19]. FgSwi6 is required for cellulose utilization in the filamentous fungus *Fusarium graminearum* [20]. Deletion of MoSwi6 caused a significant reduction in the transcription and activities of extracellular enzymes including peroxidases and laccases in *Magnaporthe oryzae* [21]. However, it is unknown whether Swi6 in *G. lucidum* is involved in cellulose degradation, and the relevant mechanism needs further analysis.

This study provides a detailed characterization of the involvement of GlSwi6 in the regulation of cellulase and xylanase activity. The putative transcription factors CreA, AceI, and AmyR were identified to play negative roles in GlSwi6-regulated cellulase synthesis. Furthermore, Ca^2+^ signaling was found to play a crucial role in the cellulase and xylanase activities regulated by GlSwi6. These results provide insight for further improvement of cellulase or xylanase activities in *G. lucidum* as well as other fungi.

## 2. Materials and Methods

### 2.1. Fungal Strains and Culture Conditions

*G. lucidum* ACCC53264 used as the wild-type (WT) strain in this study was obtained from the Agricultural Culture Collection of China. The GlSwi6-silenced strains (Swi6D and Swi6E), SiControl1 and SiControl2 (the empty vector controls), and GlSwi6-overexpression strains (OE6A-18, OE6A-25, OE6B-3, and OE6B-8) were constructed previously [22] and cultured on CYM medium (1% maltose, 2% glucose, 0.2% yeast extract, 0.2% tryptone, 0.05% MgSO_4_•7H_2_O, and 0.46% KH_2_PO_4_) at 28 °C. Fermentation experiments were performed in CYM medium using the culture shaker (Hualida Laboratory Equipment, Taicang, China), as previously described [23].

### 2.2. Enzymatic Activity Assays

Cellulase was induced using cellulose as the sole carbon source through a two-stage cultivation strategy according to a previously described method [24]. The WT, SiControls, GlSwi6-silenced, and GlSwi6-overexpressing strains were first cultured in CYM medium with shaking for 5 days at 28 °C. Then, the cultures were collected and washed thoroughly using 0.85% (*w/v*) NaCl and transferred to modified MCM medium (1% cellulose, 0.05% MgSO_4_•7H_2_O, 0.46% KH_2_PO_4_, 0.5% (NH_4_)_2_SO_4_ and 2 mL/L trace element) containing 1% (*w/v*) cellulose as the sole carbon source with shaking for 2 days at 28 °C. The CaCl_2_ (5 mM), LaCl_3_ (5 mM) and EGTA (5 mM) were added when cultures were transformed into MCM medium. The culture supernatants were collected for the detection of endoglucanase (CMCase) and xylanase activities with a DNS reagent (10 g/L 3, 5-dinitrosalicylic acid, 20 g/L sodium hydroxide, 200 g/L sodium potassium tartrate, 2 g/L redistilled phenol, and 0.5 g/L sodium sulfite anhydrous) against 1% (*w/v*) carboxymethylcellulose sodium salt (CMC-Na) and 1% (*w/v*) xylan. The mixture was incubated overnight. The following components were added to a 2.0 mL reaction mixture (containing 0.5 mL diluted culture supernatants and 1.5 mL CMC-Na or xylan solution, respectively), and then transferred into a 25 mL tube to detect activities of CMCase or xylanase. They were mixed gently and then the reaction mixture was incubated at 50 °C for 30 min. Three milliliters of DNS reagent were added to stop the reaction. A blank tube (with boiled crude enzyme) was used as a control to correct for the presence of reducing sugars in the crude enzyme samples. The blank tube was placed in boiling water for 10 min, 20 mL of distilled water and 200 μL of reaction mixture were added, and the absorbance was detected at 540 nm with the spectrophotometer (UV-1800, Shimadzu Corporation, KYOTO, Japan). One unit of enzyme activity was defined as the amount of enzyme required to release 1 μmol of glycoside bonds of the substrate per minute under defined assay conditions. Three independent cultures were taken for analysis.

### 2.3. Free Cytosolic Ca^2+^ Labeling and Detection

The levels and localization of free cytosolic Ca^2+^ in *G. lucidum* strains were monitored with a membrane-permeable compound, Fluo-3 AM (F-1241, Life Technologies Corporation, USA), according to a previous method [25,26]. Mycelia were stained with Fluo-3AM (50 μM) and incubated for 30 min, and then the residual stain was washed with phosphate-buffered saline (PBS) for the final detection with a confocal laser scanning microscope TCS SP2 (Leica, Heidelberg, Germany). The unlabeled Fluo-3AM control cells were also monitored as a control to eliminate the contribution of background fluorescence.

### 2.4. RNA Extraction and Quantitative Real-Time PCR (qRT-PCR)

Mycelia samples of *G. lucidum* cultivated in the corresponding medium were used for RNA extraction. Total RNA was extracted using RNAiso™ Plus Reagent (TaKaRa, Dalian, China) according to a previously described method [27]. cDNA was synthesized from total RNA using a Prime-Script RT Reagent Kit (TaKaRa) according to the manufacturer′s instructions. The levels of gene-specific mRNAs in WT and transformant strains were assessed using qRT-PCR with Eppendorf Mastercycler ep Realplex 2.2 software (Eppendorf, Hamburg, Germany), and 18 S rRNA was used as the internal reference gene. Transcriptional levels of genes in this study were analyzed using the standard curve method and were normalized against the 18 S rRNA gene levels. The transcription levels of all genes were expressed as relative expression levels. The primers used for qRT-PCR are listed in Supplementary Table S1.

### 2.5. Statistical Analysis

Statistical analysis was carried out using GraphPad Prism version 6.0 (GraphPad Software, San Diego, CA, USA (accessed on 10 February 2022). Data averaged from three individual experiments are expressed as the mean ± standard error (SE). Error bars present the standard deviations from the means of triplicates. The significant differences between analyzed samples were determined by Tukey′s honestly significant difference test (HSD, *p* < 0.05) and indicated by different letters.

## 3. Results

### 3.1. Effect of GlSwi6 on Cellulase and Xylanase Activities

**Figure 1:**
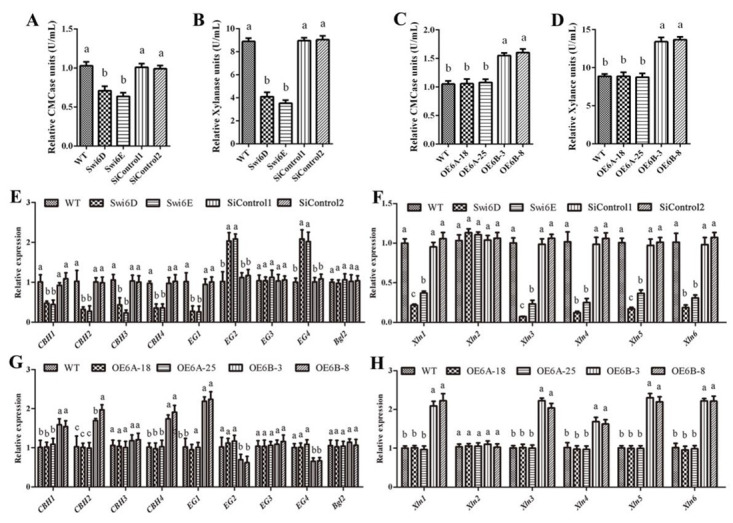
Effect of GlSwi6 on cellulase and xylanase activities. (**A**,**C**) CMCase activities in GlSwi6-silenced (**A**) and overexpression strains (**C**). (**B**,**D**) Xylanase activities in GlSwi6-silenced (**B**) and overexpression strains (**D**). (**E**,**G**) The expression of cellulase genes in GlSwi6-silenced (**E**) and overexpression strains (**G**). (**F**,**H**) The expression of xylanase genes in GlSwi6-silenced (**F**) and overexpression strains (**H**). *G. lucidum* cultured in CYM medium for 5 days was transferred to MCM medium using cellulose as a sole carbon source for 24 h. Values are the mean ± SE (*n* = 3). Different letters indicate significant differences between the strains (*p* < 0.05, Tukey′s test).

To investigate the influence of GlSwi6 on cellulase production, we first assessed the cellulase and xylanase activities of GlSwi6-silenced strains. As shown in Figure 1A, the cellulase activities of the GlSwi6-silenced strains, Swi6D and Swi6E, were 69% and 62% of those of the WT strain, respectively (Figure 1A). Besides, since xylanase is the major hemicellulose enzyme, we used xylanase to measure hemicellulose activities. It was found that xylanase activity was also decreased by approximately 54% and 60% in Swi6D and Swi6E compared with that of the WT strain (Figure 1B). The transcript levels of genes related to cellulase and xylanase were also examined. Cellulases include cellobiohydrolases (CBH, encoded by *cbh1*, *cbh2*, *cbh3*, *cbh4*), endoglucanases (EG, encoded by *eg1*, *eg2*, *eg3*, *eg4*) and beta-glucosidases (BGL, encoded by *bgl2*). Xylanase include xylanase genes (encoded by xln1, xln2, xln3, xln4) in *G. lucidum*. Among these genes, four *cbh* genes (*cbh1*, *cbh2*, *cbh3,* and *cbh4*), one *eg* gene (*eg1*), and five *xln* genes (*xln1*, *xln3*, *xln4*, *xln5,* and *xln6*) were significantly decreased in the GlSwi6-silenced strains compared with the WT strain (Figure 1E,F). These results suggest that silencing of GlSwi6 leads to a decrease in cellulase and xylanase activities in *G. lucidum*.

To further analyze the role of GlSwi6 in cellulase production in *G. lucidum*, we tested the cellulase and xylanase activities of the GlSwi6 overexpression strains. Our previous study found that GlSwi6 is alternatively spliced to generate two isoforms, Glswi6A and Glswi6B [22]. To clarify the effect of GlSwi6 on cellulase production. The GlSwi6A- and GlSwi6B-overexpressing strains were used here for further investigation. As shown in Figure 1, the cellulase and xylanase activities of the GlSwi6B overexpression strains (OE6B-3 and OE6B-8) were increased by approximately 50% and 60%, respectively, compared with those of the WT strain, respectively (Figure 1C,D). Consistently, the expression of three *cbh* genes (*cbh1*, *cbh2*, and *cbh4*), five *xln* genes (*xln1*, *xln3*, *xln4*, *xln5,* and *xln6*), and *eg1* was upregulated in the GlSwi6B overexpression strains (OE6B-3 and OE6B-8) compared with those of the WT strain (Figure 1G,H). However, the enzyme activities and gene expression levels of the OE6A-18 and OE6A-25 strains did not differ from those of the WT. These results indicate that overexpression of GlSwi6B increased the activities of cellulase and xylanase in *G. lucidum*.

### 3.2. The Effect of GlSwi6 on the Expression of Cellulase and Xylanase Regulator Genes

**Figure 2:**
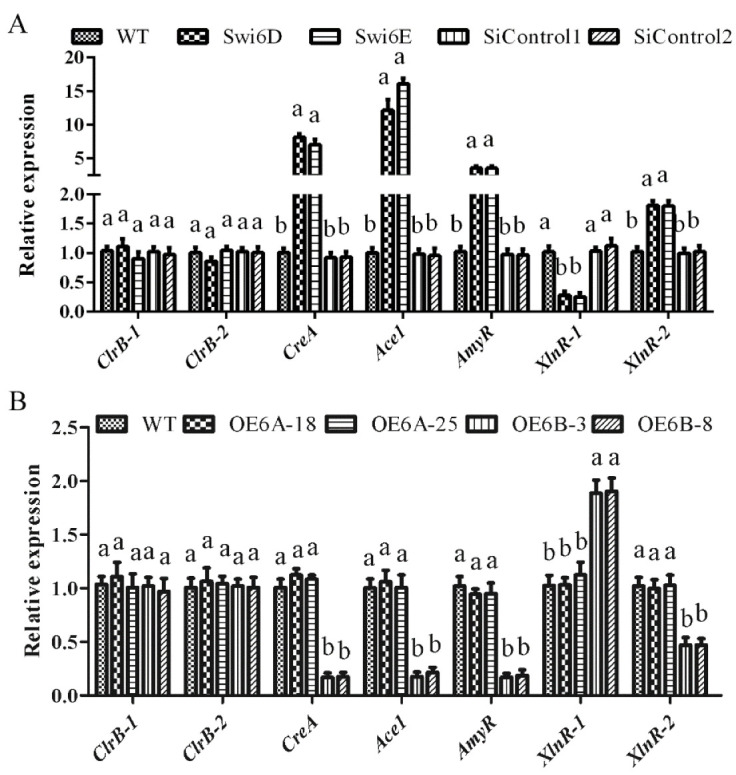
GlSwi6 is involved in regulating the expression of cellulase and xylanase regulator genes. *G. lucidum* cultured in CYM liquid medium for 5 days was transferred to MCM medium using cellulose as the sole carbon source for 24 h. (**A**) The expression of cellulase and xylanase regulator genes in GlSwi6-silenced strains. (**B**) The expression of cellulase and xylanase regulator genes in GlSwi6 overexpression strains. The values are the mean ± SE (*n* = 3). Different letters indicate significant differences between the strains (*p* < 0.05, Tukey′s test).

To further explore the influence of GlSwi6 silencing or overexpression on cellulase and xylanase activities, we explored the expression of cellulase and xylanase regulator genes in GlSwi6 silencing and overexpression strains. The expression of three cellulolytic regulator genes (*creA*, *ace1,* and *amyR*) exhibited significant differences in GlSwi6-silenced strains and GlSwi6B overexpression strains (OE6B-3 and OE6B-8) compared with that of the WT strain. As shown in Figure 2A, silencing of GlSwi6 induced a significant increase of approximately 7.59-fold in the expression of the *creA* gene, 14.13-fold in the *ace1* gene, and 3.52-fold in the *amyR* gene compared with the WT strain. Accordingly, the expression of these genes in the OE6B-3 and OE6B-8 strains decreased to 16.67–21.33% of that in the WT strain. In addition, one of the xylanase regulator genes (*xlnR-1*) was downregulated to approximately 26.83% in the GlSwi6-silenced strains and was upregulated approximately 1.89-fold in the GlSwi6B overexpression strains (OE6B-3 and OE6B-8) compared with the WT strain, while another xylanase regulator gene (*xlnR-2*) was upregulated in the GlSwi6-silenced strains and was downregulated in GlSwi6B overexpression strains (OE6B-3 and OE6B-8) compared with that of the WT strain (Figure 2). In addition, the expression of the cellulolytic regulatory genes *clrB-1* and *clrB-2* showed no significant difference in either the GlSwi6-silenced strains or the GlSwi6B overexpression strains compared with the WT strain. These results indicate that GlSwi6 differentially influences the expression of most cellulolytic regulator genes and xylanase regulator genes in *G. lucidum*. Among them, GlSwi6 exhibited a significant negative correlation with three negative regulators, while positively regulating the *xlnR-1* gene. It is speculated that GlSwi6 might indirectly regulate the expression of the *xlnR-1* gene. Taken together, these results suggest that GlSwi6 positively regulates the expression of cellulase and xylanase regulator genes.

### 3.3. GlSwi6 Regulates the Cytosolic Ca^2+^ Content

**Figure 3:**
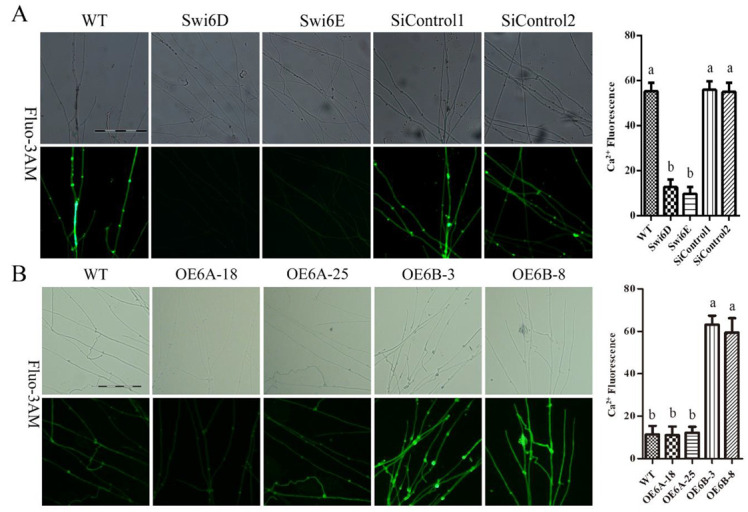
Cytosolic Ca^2+^ content in GlSwi6-silenced and overexpression strains. The GlSwi6-silenced strains were cultured on CYM plates at 28 °C for 5 ~ 7 days. (**A**) The Fluo-3AM staining (scale bar = 0.1 mm). (**B**) Analysis of Ca^2+^ fluorescence. The values are the mean ± SE (*n* = 3). Different letters indicate significant differences between the strains (*p* < 0.05, Tukey′s test).

Previous studies in *G. lucidum* indicated that Ca^2+^ plays an important role in cellulase production [15]. Therefore, we measured the cytosolic Ca^2+^ level in the GlSwi6*-*silenced and overexpression strains using the Ca^2+^-fluorescent probe Fluo-3AM. As shown in Figure 3A, the intensity of Ca^2+^ fluorescence in GlSwi6-silenced strains was significantly decreased compared with that of the WT strain. Moreover, the transcriptional regulation of Ca^2+^ signaling by GlSwi6 was further examined, including calmodulin (*Cam*), calcineurin (*Cna*), calcineurin-responsive zinc (*Crz*) finger transcription factor, Ca^2+^-permeable channel (*Mid*), phospholipase C (*Plc*), and three Ca^2+^ pumps (*GL21462*, *GL21619,* and *GL28993*) [27]. The results showed that the expression of *Cna*, *Crz*, *Plc*, *GL21462,* and *GL21619* in the GlSwi6-silenced strains was significantly downregulated compared with that in the WT strain (Figure S1A). In addition, the Ca^2+^ fluorescence intensity of the GlSwi6B overexpression strains (OE6B-3 and OE6B-8) was increased, and the expression levels of *Cna*, *Plc*, and *GL21619* in the GlSwi6B overexpression strains (OE6B-3 and OE6B-8) were upregulated compared with those in the WT strain (Figure S1B). These results suggest that GlSwi6 positively regulates the intracellular Ca^2+^ content in *G. lucidum*.

### 3.4. The Cytosolic Ca^2+^ Content Affects the Regulation of Cellulase and Xylanase Activities by GlSwi6

**Figure 4:**
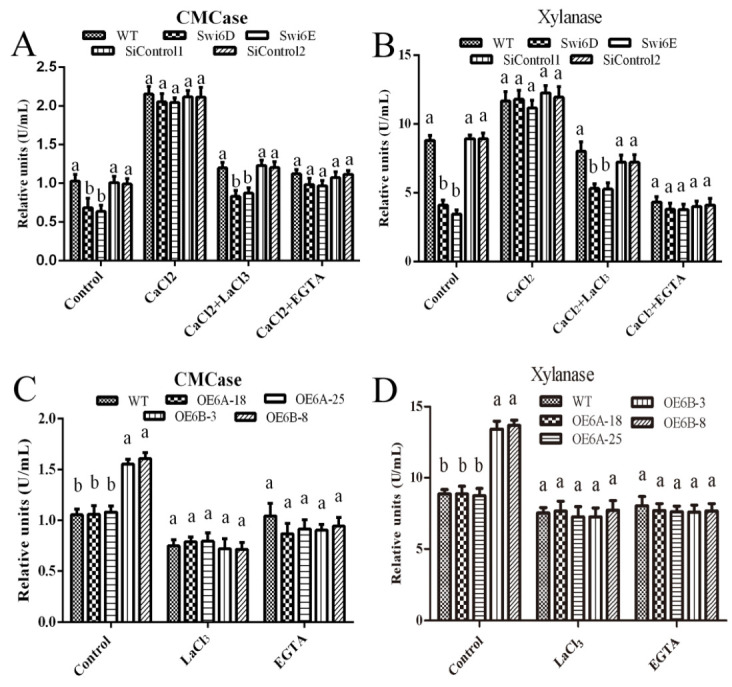
Intracellular Ca^2+^ content is involved in the regulation of cellulase and xylanase activity by GlSwi6. *G. lucidum* cultured in CYM liquid medium for 5 days were transferred to MCM medium using cellulose as the sole carbon source and then treated with 5 mM CaCl_2_, LaCl_3_, or EGTA, respectively for 24 h. The CMCase activities in GlSwi6-silenced strains (**A**,**C**) and GlSwi6 overexpression strains (**B**,**D**). The values are the mean ± SE (*n* = 3). Different letters indicate significant differences between the strains (*p* < 0.05, Tukey′s test).

We further explored whether changing the Ca^2+^ content could alter the cellulase and xylanase activities in the GlSwi6 transformants. As shown in Figure 4, silencing GlSwi6 decreased the activities of cellulase and xylanase; however, the decreased cellulase and xylanase activities in GlSwi6-silenced strains could be recovered to the levels in WT strains after the addition of 5 mM CaCl_2_. Furthermore, Ca^2+^ antagonists (LaCl_3_ and EGTA) were used to confirm the effects of Ca^2+^ content on the cellulase and xylanase activities in GlSwi6-silenced strains. As expected, the activities of cellulase and xylanase in GlSwi6-silenced strains were decreased again after treatment with 5 mM CaCl_2_ plus 5 mM LaCl_3_ or 5 mM EGTA (Figure 4A,B). In addition, the upregulation of cellulase and xylanase activities in the GlSwi6B overexpression strains (OE6B-3 and OE6B-8) could also be recovered to that in the WT strain when treated with 5 mM LaCl_3_ or 5 mM EGTA (Figure 4C,D). These results demonstrated that GlSwi6 enhanced the activities of cellulase and xylanase through the Ca^2+^ signal.

### 3.5. Effects of Ca^2+^ on the Expression Levels of Cellulolytic Regulator Genes

**Figure 5:**
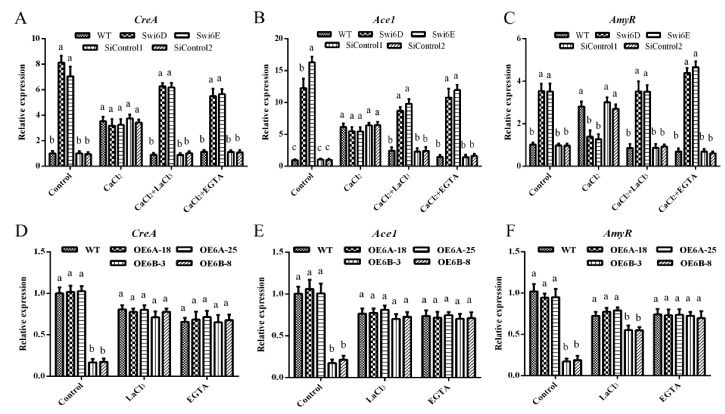
Intracellular Ca^2+^ is involved in regulating the expression of *CreA*, *Ace1*, and *AmyR* by GlSwi6. *G. lucidum* cultured in CYM liquid medium for 5 days were transferred to MM medium using cellulose as the sole carbon source and then treated with 5 mM CaCl_2_, LaCl_3_, or EGTA, respectively, for 24 h. (**A**,**D**) The expression of *CreA* in GlSwi6-silenced strains and GlSwi6 overexpression strains. (**B**,**E**) The expression of Ace1 in GlSwi6-silenced and overexpression strains. (**C**,**F**) The expression of *AmyR* in GlSwi6-silenced and overexpression strains. The values are the mean ± SE (*n* = 3). Different letters indicate significant differences between the strains (*p* < 0.05, Tukey′s test).

To further analyze the effect of Ca^2+^ on cellulase and xylanase activities, we explored the influence of the Ca^2+^ on the expression of cellulolytic regulator genes. A previous study indicated that the expression of *CreA*, *Ace1,* and *AmyR* varied significantly compared to that of other genes, so we tested the expression of these three genes when treated with CaCl_2_ or a Ca^2+^ antagonist. (Figure 5). Consistent with the changes in cellulase and xylanase activity, the expression levels of *CreA*, *Ace1,* and *AmyR* genes were significantly upregulated in GlSwi6-silenced strains. When treated with 5 mM CaCl_2_, the trend of the upregulation disappeared, and the addition of 5 mM CaCl_2_ plus 5 mM LaCl_3_ or 5 mM EGTA rescued the upregulation in these genes in GlSwi6-silenced strains (Figure 5A–C). In addition, the downregulated of the three genes (*CreA*, *Ace1,* and *AmyR*) in GlSwi6B overexpression strains were also recovered when treated with 5 mM LaCl_3_ or 5 mM EGTA compared with those of the control strains (Figure 5D–F). Taken together, these results indicate that the cytosolic Ca^2+^ content significantly promotes the transcriptional effect of GlSwi6 on cellulolytic regulator genes.

## 4. Discussion

Current studies on the regulation of cellulose degradation focused on the following aspects, enzymes related to cellulose degradation [28], and the regulation of degradation genes by transcription factors [29,30,31]. In addition, signaling factors have also been found to play an important role in this process [32]. The regulation of cellulases has been extensively studied in *T. reesei* [10]. As an increasing number of cellulose synthesis-related gene functions have been discovered, the process of cellulase synthesis is becoming clearer. The composition of cellulase-related genes has been systematically studied, and all these genes belong to the GH family. However, positions of lignocellulolytic enzymes-related genes are not closely clustered in the genome according to the study by Chen [2], referring to the scaffold information in the genomic data, that only *cbh1*, *cbh3*, *eg1*, and *xlnR-1* were clustered in scafford1 and *amyR* clustered with *creA* in scafford29. Furthermore, detection of transcript levels of genes encoding CBH, EG, and XLN showed that GlSwi6 positively regulated the expression of *cbh* genes and most *xln* genes. This suggests that GlSwi6 is involved in regulating the production of cellulase and xylanase. In addition to these genes, transcription factors also play an irreplaceable role in this process. Recent studies in *T. reesei* found Xyr1 in functions as the central regulator of cellulases to control the expression of CAZyme genes by interacting with TrGAL11 [33]. However, the expression of *xyr1* was negatively regulated by the CCR proteins Cre1 and Ace1 [34]. Therefore, the identification of transcription factors involved in cellulases, tuning the positive regulators, is a promising strategy to enhance cellulase expression in fungi. Our study found that GlSwi6 negatively regulated three cellulolytic regulator genes (*creA*, *ace1*, and *amyR*), and these genes play a negative regulatory role in cellulase synthesis. Therefore, the positive effect of GlSwi6 on cellulase and xylanase was further confirmed.

Environmental signaling also causes alterations in the expression of downstream target genes through modifications of transcription factors. To date, the light-mediated response, secondary messenger cyclic AMP (cAMP), MAPK pathways and the Ca^2+^-responsive signaling pathway have been found to mediate cellulase expression [10]. Our study in *G. lucidum* also demonstrated that MAPK pathways are involved in the regulation of cellulase and xylanase activities. Among them, MAPK pathways are the best-characterized signaling pathways in eukaryotes and their functions are conserved in fungi [35]. All *T. reesei* MAPKs were reported to be involved in the cellulase formation, and the yeast FUS3-like Tmk1 and yeast Slt2-like Tmk2 repressed cellulase formation [35,36], and found an indirect connection between cellulase production and MAPK [37]. However, the specific mechanism has not been elucidated. Our previous studies reported that the role of GlSlt2/MAPK and its downstream target GlSwi6 are involved in fungal growth and fruiting body development [23]. Here, we found that GlSwi6 positively regulates the cellulases and xylanases activities in *G. lucidum*. This finding has important implications for further explaining the mechanism by which the MAPK signaling pathway regulates cellulose degradation.

Studies on the signals involved in the regulation of cellulose degradation are limited thus far. The role of the Ca^2+^-responsive signaling pathway was shown to play a role in regulating the expression and secretion of cellulase in *Trichoderma reesei* Rut-C30; the calcineurin-responsive zinc finger transcription factor Crz binding to the promoters of Cbh1 and Xyr1 in response to Ca^2+^ [14]. Further study also found that NAD+ mediated the regulation of cellulase genes through modulating cytosolic Ca^2+^ [15]. Ca^2+^ signaling and the MAPK signaling pathways have been found to play an essential role in this process [38]. However, the connection between Ca^2+^ signaling and the MAPK signaling pathway is still unclear. Analysis of the interactions between different signals contributes to a comprehensively understanding of the regulatory network of cellulase and xylanase. Our study found that GlSwi6 positively regulates cellulase and xylanase activities in Ca^2+^ signaling. Furthermore, we also found that GlSwi6 increased cytosolic Ca^2+^ content in *G. lucidum*. Analysis of the gene expression of Ca^2+^ signaling showed that genes upstream of Ca^2+^ signaling (*plc* and *GL21619*) were highly activated by GlSwi6. This demonstrated that GlSwi6 positively regulated the influx of Ca^2+^. Furthermore, pharmacological experiments, such as exogenous addition of CaCl_2_ and the Ca^2+^ antagonist LaCl_3_, demonstrated that the Ca^2+^ promotes the regulation of cellulase and xylanase activities by GlSwi6. This phenomenon is similar to that in the study by Wang [15]. However, the specific regulatory mechanism of GlSwi6 on cellulase and xylanase activities is still unclear.

It was reported that Swi6 plays essential regulatory functions in cell growth and development, which include the cell cycle and cell wall integrity [39,40]. In the present study, Swi6 was found to have a regulatory effect on cellulase and xylanase activities, which is similar to previous studies; Swi6 is an important regulator of cellulose decomposition and utilization in *Fusarium graminearum* [20]. GlSwi6 was reported to be alternatively spliced into two different variants, GlSwi6A and GlSwi6B, and GlSwi6B plays a crucial role in the cell wall integrity in *G. lucidum* [22]. In this study, we also found that GlSwi6B increases cellulase and xylanase activities, while GlSwi6A shows no effect on these activities. This implies that the different variants of GlSwi6 might exhibit different physiological functions in *G. lucidum*, which requires further exploration.

In summary, our study reveals the mechanism of the Slt2/MAPK-GlSwi6-Ca^2+^ signaling pathway underlying cellulose degradation in *G. lucidum*. We conducted a detailed analysis of the progress about Ca^2+^ signaling and MAPK pathways involved in this study, and the regulation of cellulase by these signaling pathways was also found in *G. lucidum*. We further found that the transcription factor GlSwi6 plays an important role in these two pathways. Combined with relevant progresses and our results, we came to a novel conclusion that the Ca^2+^ signaling cross talk with MAPK pathways to regulate cellulase production. Our study connects different signaling pathways together to form a synergistic regulatory network. This also provides guidance for future integrated effects of different cellulose degradation processes.

## Data Availability

Not applicable.

## Supplementary Materials

The following supporting information can be downloaded at: https://www.mdpi.com/article/10.3390/jof8020187/s1, Figure S1: GlSwi6 regulated the expression of genes involved in Ca^2+^ signal; Table S1: Primers used in this study.
