# Supplementary material for: GlSwi6 Positively Regulates Cellulase and Xylanase Activities through Intracellular Ca2+ Signaling in Ganoderma lucidum
Source: J Fungi (Basel). 2022 Feb 14;8(2):187. doi: 10.3390/jof8020187 (PMC8877461; doi:10.3390/jof8020187)
Supplement: Supplementary file 1 [file jof-08-00187-s001.zip › jof-1594008-supplementary.pdf]

Supplementary information

GlSwi6 is involved in the regulation of cellulase and xylanase activities through intracellular Ca^{2+} signaling in *Ganoderma lucidum*

Ling-Dan Lian,[1] Ling-Yan Shi,[1] Jing Zhu,[1] Rui Liu,[1] Liang Shi,[1] Ang Ren,[1] Han-Shou Yu[1] and Min-Wen Zhao[1]*

Affiliation:

[1]Key Laboratory of Agricultural Environmental Microbiology, Ministry of Agriculture, Microbiology Department, College of Life Sciences, Nanjing Agricultural University, Nanjing, Jiangsu, 210095, China.

*Corresponding author. Mailing address: College of Life Sciences, Nanjing Agricultural University; No.1 Weigang, Nanjing 210095, Jiangsu, People′s Republic of China.

Telephone and Fax: 0086-25-84395602.

E-mail: mwzhao@njau.edu.cn

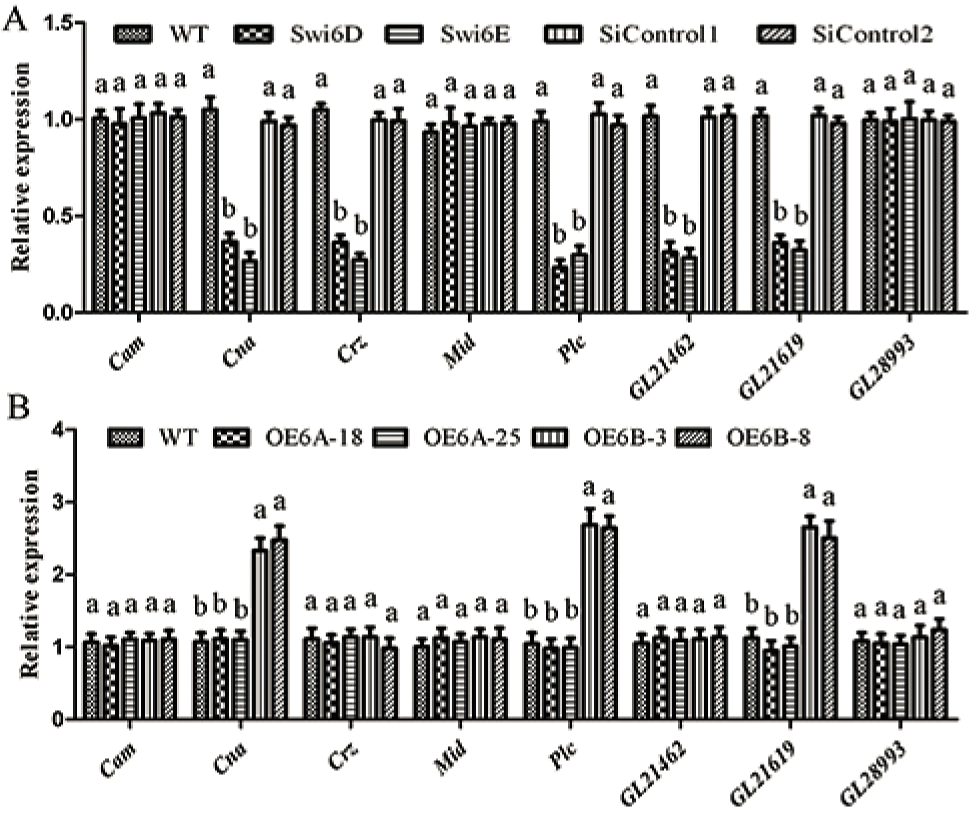

Fig.S1 GlSwi6 regulated the expression of genes involved in Ca^{2+} signal. (A) The expression of genes involved in Ca^{2+} signal in *GlSwi6* silenced strains. (B) The expression of genes involved in Ca^{2+} signal in *GlSwi6* overexpression strains. The values are the mean ± SE (n=3). Different letters indicate significant differences between the strains ($P < 0.05$, Tukey′s test).

Table S1 Primers used in this study

Primer	Sequence (5′to 3′)	Description
RT-CBH1-F	ACGCTCACCACCGAGAC	Detects the CBH1 expression
RT-CBH1-R	CGTAGTCAGCACCATCCAG	
RT-CBH2-F	ACTGGACTGGCTCTGCTAACG	Detects the CBH2 expression
RT-CBH2-R	ATCGCAGCCGTCCTTGTCG	
RT-CBH3-F	GCTTTGCCCTGACCCTGTC	Detects the CBH3expression
RT-CBH3-R	CGTAGGTGGTGTCATCCTGTGA	
RT-CBH4-F	CGCGTCTGGCTCAGTTTG	Detects the CBH4 expression
RT-CBH4-R	CAGCGTAGTATGGGCTCAAGT	
RT-EG1-F	CTCCGTCCAATTTGTCGT	Detects the EG1 expression
RT-EG1-R	GTTCCACTGTTGTCGCTGT	
RT-EG2-F	ACGGTTCAATGGCGAGATAA	Detects the EG2 expression
RT-EG2-R	CGCACCTGCTTGACGAAT	
RT-EG3-F	CACCGACTCACTCACCCTCA	Detects the EG3 expression
RT-EG3-R	CGCGGACACCGTTCACA	
RT-EG4-F	GTTCCTCGCTTTGTCAG	Detects the EG4 expression
RT-EG4-R	CACGAATTCAGGGTCGC	
RT-Bgl2-F	ACATCTCTCTTCCCGAC	Detects the Bgl2 expression
RT-Bgl2-R	TATCCACACTGCTCCTG	
RT-Xln1-F	GCAGACGCCTTGTTAGC	Detects the Xln1 expression
RT-Xln1-R	AGCCTCGGGAGTGTTGA	
RT-Xln2-F	CTCCCCTCACAACTACAC	Detects the Xln2 expression
RT-Xln2-R	CATACAAGGACGACAAAA	
RT-Xln3-F	GAACGCTGATGTTTGGC	Detects the Xln3 expression
RT-Xln3-R	CCTTTCCGTCTCTGTCTC	
RT-Xln4-F	GCTTATCGACGCCATCG	Detects the Xln4expression
RT-Xln4-R	TCACGCACACCCCAACT	
RT-Xln5-F	CCAGTGCCACTTCATCG	Detects the Xln5 expression
RT-Xln5-R	ATACCCACACACCCCCC	
RT-Xln6-F	ACATGCGCGAGTTCGTG	Detects the Xln6 expression
RT-Xln6-R	AAGGTGCTGGGGACAAA	
RT-XlnR-1-F	GGGCTCCATCGATCTTAC	Detects the XlnR-1 expression
RT-XlnR-1-R	CCTTGCGCTTATTCTTGT	
RT-XlnR-2-F	CCCCACTCGCCTCTTGAC	Detects the XlnR-2 expression
RT-XlnR-2-R	AGCCGGAGCACTTTGCCT	
RT-ClrB-1-F	CGTCACATCTGCGTCTA	Detects the ClrB-1 expression
RT-ClrB-1-R	TTCCGTACTGATCTTGG	
RT-ClrB-2-F	CCAGAAGAATGGGAAGTC	Detects the ClrB-2 expression
RT-ClrB-2-R	GGATATGAGGTTGGTGAG	
RT-CreA-F	GACATTCCCGCATACACA	Detects the CreA expression
RT-CreA-R	GCTCCTAGCCTTCTTCTTA	
RT-Ace1-F	GGGCTATACGACTTTCGG	Detects the Ace1 expression

RT-Ace1-R	ATTGGGGTCTATGCTGGG	
RT-AmyR-F	CCAGCCCAAGAAGAGAGG	Detects the AmyR expression
RT-AmyR-R	GGGAGAGCCGAAACAGAC	
RT-Cam-F	CCCCGAGTTCCTGACGATG	Detects the Cam expression
RT-Cam-R	AGCTTCTCGCCGAGGTTGG	
RT-Cna-F	AGGAGGCTATCTCTGGTTTCG	Detects the Cna expression
RT-Cna-R	TCTTCTGCTGGCGTTGTCG	
RT-Crz-F	ACGCCCCTTCCTATGCGAGTG	Detects the Crz expression
RT-Crz-R	AGGAAACGGCGTCAGTAGC	
RT-Mid-F	CAACTTGTCCTTGCCATCAC	Detects the Mid expression
RT-Mid-R	GCCAGCCATCCGTATTCTT	
RT-Plc-F	CAACTTTGACGACGTAGAGC	Detects the Plc expression
RT-Plc-R	GGCGTGCCTTGAGGGACTT	
RT-GL21462-F	TGTCACCCACGAACCCTTAT	Detects the Ca^{2+} pump expression
RT-GL21462-R	GGAGGACCTTTGCTAGACG	
RT-GL21619-F	TGACGAAGAACGAGCAGAC	Detects the Ca^{2+} pump expression
RT-GL21619-R	TGTTGCAGACCGAGCCTATC	
RT-GL28993-F	CGCGAATGTCCTCCCTACT	Detects the Ca^{2+} pump expression
RT-GL28993-R	GCAACACCTTCCACCCAAT	
